# Two-phase and family-based designs for next-generation sequencing studies

**DOI:** 10.3389/fgene.2013.00276

**Published:** 2013-12-13

**Authors:** Duncan C. Thomas, Zhao Yang, Fan Yang

**Affiliations:** Department of Preventive Medicine, University of Southern CaliforniaLos Angeles, CA, USA

**Keywords:** sequencing, two-phase sampling design, family-based study, rare variant association, breast neoplasms, colorectal cancer

## Abstract

The cost of next-generation sequencing is now approaching that of early GWAS panels, but is still out of reach for large epidemiologic studies and the millions of rare variants expected poses challenges for distinguishing causal from non-causal variants. We review two types of designs for sequencing studies: two-phase designs for targeted follow-up of genomewide association studies using unrelated individuals; and family-based designs exploiting co-segregation for prioritizing variants and genes. Two-phase designs subsample subjects for sequencing from a larger case-control study jointly on the basis of their disease and carrier status; the discovered variants are then tested for association in the parent study. The analysis combines the full sequence data from the substudy with the more limited SNP data from the main study. We discuss various methods for selecting this subset of variants and describe the expected yield of true positive associations in the context of an on-going study of second breast cancers following radiotherapy. While the sharing of variants within families means that family-based designs are less efficient for discovery than sequencing unrelated individuals, the ability to exploit co-segregation of variants with disease within families helps distinguish causal from non-causal ones. Furthermore, by enriching for family history, the yield of causal variants can be improved and use of identity-by-descent information improves imputation of genotypes for other family members. We compare the relative efficiency of these designs with those using unrelated individuals for discovering and prioritizing variants or genes for testing association in larger studies. While associations can be tested with single variants, power is low for rare ones. Recent generalizations of burden or kernel tests for gene-level associations to family-based data are appealing. These approaches are illustrated in the context of a family-based study of colorectal cancer.

## Introduction

In the early days of genomewide association studies (GWAS), the cost of commercial high-density genotyping panels was prohibitive for large-scale epidemiologic studies needed to detect the modest relative risks (RRs) now known to be associated with most common variants for complex diseases (Hindorff et al., 2009). Hence, investigators turned to multi-stage designs, in which only a sample of subjects were genotyped on such platforms and a generous selection of the most significant associations were then tested on an independent sample using custom genotyping techniques. The final analysis typically combined the data from both stages, with a final significance level chosen to ensure genomewide significance after allowing for the number of variants tested in the second. The basic principles were based on a series of papers written before the GWAS era (Satagopan et al., 2002, 2004; Satagopan and Elston, 2003), and subsequent work showed how to optimize the allocation of sample size and first-stage critical values in the GWAS context (Wang et al., 2006; Skol et al., 2007). In particular, Skol et al. (2006) showed that this joint analysis was more powerful than treating the design as discovery followed by independent replication, despite various high-profile journals' requirements for an independent replication study (Panagiotou et al., 2012). Although this became the conventional GWAS design throughout the first decade of the 21st century, rapidly declining costs of commercial GWAS chips have made it feasible for many studies to obtain genome-wide coverage on *all* available subjects in a single stage (Thomas et al., 2009a,b). The cost of custom genotyping for large numbers of hand-picked SNPs was often comparable to standard high-density panels, and having more subjects with genome-wide data allowed for more informative analysis of interactions, subgroups, pleiotropic effects, etc. For a general review of multi-stage designs in genetics, see (Elston et al., 2007).

As we entered the “post-GWAS” era, the focus began to shift toward rare variants and the use of next-generation sequencing (NGS) technologies that could in principle (given a large enough sample size and deep enough sequencing) uncover *all* the genetic variation in a region, not just the common SNPs that have been used to tag the unknown causal variants. In part, this interest stemmed from the increasing recognition that common variants were accounting for only a relatively small proportion of the total heritability of most complex diseases (Manolio et al., 2009; Schork et al., 2009). Amongst other possible explanations for the “missing heritability,” rare variants have been proposed, based on an evolutionary argument (Gorlov et al., 2011) or empirical evidence (Bodmer and Bonilla, 2008) that their effect sizes could be larger, although recent whole-exome sequencing studies have cast some doubt on this hypothesis (e.g., Heinzen et al., 2012). Furthermore, since rare variants tend not to be well tagged by common ones (Duan et al., 2013), use of conventional GWAS panels would tend to miss associations with rare variants. Cost currently precludes application of NGS to whole-genome sequencing on a large scale, so clever study design has again become important (Thomas et al., 2009a,b). One of the first uses of NGS was for targeted follow-up of GWAS hits, for which an alternative to two-stage designs, known as two-*phase* designs, is a natural choice. These differ from the two-*stage* designs described above in that the set of subjects chosen for expensive data collection (e.g., NGS) are a *proper subset* of a larger epidemiologic study rather than an independent sample and that this subset is selected on the basis of information already available on the full study (Whittemore and Halpern, 1997; Thomas et al., 2004; Yang and Thomas, 2011). In the case of NGS, this could involve stratification *jointly* on disease status and carrier status of the associated variant(s). While this would tend to induce a spurious association between any variants in LD with the GWAS SNPs and disease even under the null hypothesis that they are not causal, this bias can be avoided by adjusting for the sampling fractions, and additional information available in the full study can also be incorporated. The basic principles were developed in a series of seminal papers by Norman Breslow with various colleagues (see Breslow and Holubkov, 1997b; Breslow and Chatterjee, 1999; Scott et al., 2007; Breslow et al., 2009b, for summaries of this work). Recently, Schaid et al. (2013a) has provided an excellent discussion of the use of this approach for targeted follow-up of GWAS hits by NGS. However, for whole genome or whole exome sequencing studies, there would be no point in selecting individuals based on whether they carried a specific polymorphism, except to eliminate those known to be carrying a known major mutation.

Most GWAS for discovering common variants associated with disease traits have been conducted using a case-control design with unrelated controls. Not only are unrelated individuals easier to identify and enroll than are entire families (particularly multiple-case families), but the statistical efficiency for discovery or association testing per subject genotyped is typically higher using unrelated controls than using unaffected siblings or other relatives (Witte et al., 1999). However, with the growing interest in rare variants and the availability of NGS, there has been a resurgence of interest in using family-based designs (Zhu et al., 2010; Feng et al., 2011; Ionita-Laza and Ottman, 2011; Shi and Rao, 2011). Family-based designs may have other advantages that outweigh their loss of statistical efficiency. By exploiting information about co-segregation, they may be more efficient at prioritizing potentially causal variants from non-causal ones for subsequent testing for association with disease in larger samples. The ability to exploit Mendelian inheritance may also improve the imputation of rare variants in untested samples (Li et al., 2009; Cheung et al., 2013). Finally, family-based designs can exploit both between- and within-family comparisons in a two-*step* analysis for better power while being robust to bias from population stratification (Lange et al., 2003; Van Steen et al., 2005; Feng et al., 2007; Murphy et al., 2008). In this paper, we focus on the first of these advantages, using a design that sequences a subset of family members initially, ranks the discovered variants in terms of their likelihood of being associated with the trait using the phenotype information on the entire family, and then tests for association in an independent sample. In this sense, the design has elements of both two-*phase* and two-*stage* designs, in that the sequencing set is a proper subset of a larger family-based study and that an independent sample is used for replication or combined analysis.

One consequence of the new focus on rare variants is the need for novel analysis strategies, because testing associations individually with every variant would have very little power due to the large multiple comparisons penalty and their rarity. In a sample of, say, size 200, one might identify about 20 million variants. Most of these are likely to be unrelated to disease and genotyping all of them for a large case-control association study would be neither feasible nor statistically efficient, so some means of identifying those most likely to be causal is needed. Furthermore, under some models of disease causation, multiple variants in a causal gene (or pathway) could affect its function, so aggregating variants within genes may also improve power. To address this need, a host of “burden” tests have been developed based on counts of rare variants, weighted in various fashions (see Asimit and Zeggini, 2010; Cirulli and Goldstein, 2010; Basu and Pan, 2011; Bacanu et al., 2012; Thomas, 2012, for reviews). However, these are ill-suited to the situation where a region contains both deleterious and protective variants (Hoffmann et al., 2010). A random effects model that focuses instead on the *variance* of risk across variants rather than their mean might therefore be more powerful. The first of this type was the C_α_ test (Neyman and Scott, 1966; Neale et al., 2011), which tests for overdispersion of case-control ratios, conditional on the total number of variants. The Sequence Kernel Association Test [SKAT (Wu et al., 2011; Lee et al., 2012)], based on a general linear mixed model, tests for association between similarity of phenotypes and similarity of multi-locus genotypes across all pairs of subjects. See Schaid (2010a,b) for a general review of the basic statistical foundations of such tests and various choices of kernel functions for genetic applications. Recently, this class of methods has been extended to family studies (Huang et al., 2010; Schifano et al., 2012; Chen et al., 2013; Ionita-Laza et al., 2013; Schaid et al., 2013b). Hierarchical modeling approaches offer another approach to the analysis of rare variants, allowing formal incorporation of external information for prioritization.

A variety of methods for incorporating genomic context, functional, or pathway annotation data have been discussed in GWAS contexts (reviewed by Cantor et al., 2010; Thompson et al., 2013). Examples of prior information might include loci previously reported, pathway or genomic annotation, expression QTL or other functional assays, etc. (Rebbeck et al., 2004; Bush et al., 2009; Karchin, 2009; Nicolae et al., 2010; Wang et al., 2010; Freedman et al., 2011; San Lucas et al., 2012; Minelli et al., 2013). Filtering on such variables has become a popular strategy, but risks eliminating many causal variants whose potential significance has not yet been recognized or loading up the list of prioritized variants with too many non-causal ones based on irrelevant information. The weighted False Discovery Rate (Roeder et al., 2006; Wakefield, 2007; Whittemore, 2007) and Gene Set Enrichment Analysis (Chasman, 2008; Holden et al., 2008) require specification of weights in advance and there is no obvious way to combine multiple filters. The hierarchical modeling approach described below is more flexible, allowing the weights given to various biofeatures to be determined empirically, based on their observed correlation with disease associations across the ensemble of all variants.

An example of a two-phase design is the Women's Environmental Cancer and Radiation Epidemiology (WECARE) Study of the risk of second breast cancers among survivors of a first breast cancer, focusing on radiation dose to the contralateral breast (Stovall et al., 2008; Langholz et al., 2009), various genes involved in DNA damage response pathways (Begg et al., 2008; Concannon et al., 2008; Borg et al., 2010; Malone et al., 2010; Capanu et al., 2011; Quintana et al., 2011; Brooks et al., 2012; Quintana et al., 2012; Reiner et al., 2013) and their interactions (Bernstein et al., 2010, 2013); a GWAS is also currently in progress. The design is a nested case-control study, with two controls matched to each case on age and year of diagnosis of the first cancer and study center, and “counter-matched” on radiotherapy for treatment of the first cancer (Bernstein et al., 2004). As an illustration of the two-phase design, we are currently performing whole genome sequencing on a subsample of 201 subjects and whole exome sequencing on several hundred more, drawn from the 701 cases and 1399 controls, stratified jointly by case-control status and risk predictors—age at first cancer, family history (FH), radiation treatment, and time since exposure.

As an example of a family-based design, we are currently performing deep targeted resequencing of 11 replicated regions identified by previous GWASs as associated with colorectal cancer (CRC), using ~4200 samples drawn from the Colon Cancer Family Registries (C-CFR). The C-CFR is an international collaboration of registries of families ascertained through CRC in various ways, some population-based, some from high-risk genetic clinics, some including population controls or control families (Newcomb et al., 2007). To date, 10,662 CRC families have been enrolled, totaling 62,353 individuals, with genetic samples available on 5113 cases and 9196 unaffected family members or population controls with epidemiologic risk factor information, and FH data on many more (http://epi.grants.cancer.gov/CFR/about_colon.html, accessed 3/8/13). For the purpose of comparing different designs, we have selected some samples from multiple-case families and some from unrelated cases or controls in various ways. Ultimately these data would be used to compare designs empirically in terms of the yield of significant findings by subsampling from these real data (e.g., to assess whether a lower depth of sequencing, narrower regions, fewer subjects or subjects targeted in different ways would have sufficed).

The aim of this paper is to review recent developments in methods for the design and analysis of NGS studies, with a particular focus on two-phase and family-based designs, and to illustrate the various issues with simulated data and applications to power calculations and preliminary data from these two studies.

## Results

### Two-phase sampling for targeted resequencing

Suppose one has already completed a large case-control GWAS of unrelated individuals, in which one or more tag SNPs have been found to be strongly associated with a particular disease. It is unlikely that the associated SNPs would themselves be causal—more likely they are simply in LD with the truly causal variant(s). The aim of a targeted resequencing study is therefore to exhaustively re-sequence the region to identify *all* variants in the hopes of discovering these causal ones. Two key decisions are required: how to select the subsample to be sequenced; and how to prioritize the variants found in this subsample.

#### Approaches to prioritization of variants

One obvious method of prioritization would be on the basis of novelty, i.e., to focus attention on variants that have not been seen previously (or only rarely) among population controls. With the growing catalog of sequence variants in public databases like the 1000 Genomes Project, many causal variants are likely already to have been discovered and those that are novel are likely to be so rare that there would be very little power testing their association individually with disease. Furthermore, most novel variants are likely to be neutral. Nevertheless, the discovery of a novel association with disease is important, irrespective of whether or not the existence of the variant has been previously reported, but a discovery of a novel variant and its association with disease is particularly noteworthy. The same applies to filtering based on differences in allele frequencies between cases and controls within the sequencing subset (Yang and Thomas, 2011). Thus, some investigators have decided not to sequence controls, but this could be ill advised if cases are sequenced in populations not well represented in public databases or on platforms with different discovery characteristics (e.g., depth of coverage, quality control filtering).

Under the hypothesis that a gene may harbor multiple variants any of which could affect function or that a critical pathway could be affected by polymorphism in any of the genes in it, a strategy that aggregates across multiple related variants may be helpful. Methods section Simulation of Gene- and Pathway-level Prioritization in the WECARE STUDY describes a simulation of this strategy based on the WECARE study; results are discussed in the application below.

Hierarchical modeling (Greenland, 2000) entails adding a second-level model for the effect estimates of each variant, allowing the magnitude of the effects, their probability of being non-null, or their covariances to depend upon external information (“prior covariates”) (Conti and Witte, 2003; Hung et al., 2004; Chen and Witte, 2007; Hung et al., 2007; Lewinger et al., 2007; Conti et al., 2009; Hoffmann et al., 2010; Capanu and Begg, 2011; Capanu et al., 2011). Hierarchical models involve a first (subject)-level model for individual's phenotypes *Y*_*i*_ as a function of a vector of genotypes *G*_i_ = (*G*_iv_) at loci *v* and corresponding regression coefficients β = (β_v_), e.g., a general linear model of the form *f*[*E*(*Y*_*i*_)] = *G*′_*i*_β, and a second (variant)-level model for the distribution of these regression coefficients as a function of prior covariates *Z*_*v*_, e.g., a linear regression model of the form *E*(β_*v*_) = *Z*′_*v*_π (or) perhaps a model for their variances or covariances (Thomas et al., 2009a,b). This approach also has the advantage of allowing for the uncertainty about which effects should be included in the model within a Bayes model averaging framework, e.g., by modeling the *probability* that a variant has no effect as logit[Pr(β_*v*_ = 0)] = *Z*′_*v*_α (Quintana et al., 2012; Quintana and Conti, 2013). It also allows the data to determine the optimal weights for the various prior covariates rather than having to specify them *a priori*; these papers show how the gain in power from including covariates with high predictive value for classifying causality of variants is offset by very little loss of power from including covariates with low predictive value (since they are usually assigned very little weight), in contrast to filtering approaches, which can lead to substantial loss of power if either sensitivity or specificity is low.

#### Joint analysis of SNP and sequence data

The subset of subjects from the substudy with the full sequence data would probably not provide adequate power for testing associations with disease directly, either variant-by-variant or by any of the aggregation methods described above. How then might one take advantage of the data from the much larger GWAS from which the substudy subjects were selected? Three basic strategies are possible: (i) by genotyping; (ii) by imputation; or (iii) by joint analysis.

The first of these is the simplest, but most expensive. One simply does custom genotyping in the main study of the prioritized variants. Under the hypothesis that any causal variants are likely to have been discovered by sequencing and that they survived prioritization, then the genotype data for the main study should be sufficient and associations can be tested directly, with appropriate correction for the effective number of independent tests performed (Conneely and Boehnke, 2007). A final analysis can then include a test of whether the novel variants account for the original SNP association (Yang and Thomas, 2011).

Imputation has become a standard approach for GWAS analysis, so that typically several million common variant associations are tested by combining the study data from the SNP panel with population distributions of all common and uncommon variants from such databases as the HapMap and 1000 Genomes projects (Asimit and Zeggini, 2012; Howie et al., 2012). For each variant not on the GWAS panel, one computes the expected allelic dosage and uses this as the covariate in a logistic regression model; this strategy is known to be superior to simply using the most likely genotype, in part because it correctly allows for the uncertainty in the imputation (Stram et al., 2003). Whether this strategy would be viable for rare variants is still unknown, but there are two reasons for concern. First, the strategy relies on linkage disequilibrium, and rare variants tend to have weaker LD than common ones (Duan et al., 2013). Second, it also relies on having sufficiently large reference panels, which would not include newly discovered variants.

Joint analysis of the full sequence data on the subsample and the SNP data on the main study is the most powerful approach and, like imputation does not involve any further genotyping costs. In their series of seminal papers on two-phase studies, Breslow et al. describe three basic analysis approaches: pseudo-likelihood (PL), weighted likelihood (WL), and semi-parametric likelihood (Breslow and Cain, 1988; Breslow and Zhao, 1988; Cain and Breslow, 1988; Breslow and Holubkov, 1997a,b; Breslow and Chatterjee, 1999; Breslow et al., 2003, 2009a,b; Breslow and Wellner, 2007). The simplest of these is the WL approach, so for simplicity, we confine our discussion here to this one (Methods section Likelihoods for Joint Analysis of Two-phase Studies). The basic idea is based on Horvitz-Thomson estimating equations, which use the score function derived from the likelihood for a logistic regression of disease status in the substudy data alone, weighting each subject's contribution inversely by their sampling probabilities, Σ_*i*_ [*Y*_*i*_ − *p*_*i*_(β)] *W*_*i*_ *G*_*i*_ = 0, where *p*_*i*_(β) = expit(*G*′_*i*_β) and *W*_*i*_ = *N*_*si*_/*n*_*si*_, *s*_*i*_ being the sampling stratum to which subject *i* belongs and *N*_*s*_ and *n*_*s*_ the main study and substudy sample sizes respectively. While simple in concept, the disadvantage is that the only information used from the main study is the stratum sample sizes. A refinement of this approach is to replace the empiric weights based on the realized sample sizes by predicted weights based on a logistic regression of sampling probabilities on additional covariates available for all main study participants. Recent papers show how this basic approach can be stabilized by using “calibrated weights” without requiring assumptions about the validity of an imputation model using influence residuals (Breslow et al., 2009a,b). The utility of this approach for targeted follow-up of GWAS hits is discussed in Schaid et al. (2013a).

#### Optimization of sampling fractions

As with two-stage designs, it is theoretically possible to optimize the choice of sampling fractions, subject to a constraint on total cost, but in practice this requires knowledge of the true values of various model parameters (causal allele frequencies, RRs, LD with the GWAS SNPs, etc.). Fortunately, the design is often relatively insensitive to these parameters, so that a balanced design in which the various strata are represented by equal numbers *n*_*s*_ in the subsample may be nearly optimal (Reilly and Pepe, 1995; Reilly, 1996). In their article on the application of this design to targeted follow-up of GWAS hits, for example, Schaid et al. (2013a) do not address optimization, but recommend the balanced design.

As with two-stage designs, the basic idea is either to maximize power subject to a constraint on total cost or to minimize the cost required to attain a target power. If the only cost is sequencing the subsample, then it is sufficient to optimize the *proportional* allocation of substudy subjects across strata. If instead one is designing both the main study and substudy *de novo* or if custom follow-up genotyping of the main study is planned, then the *relative* sample sizes of the two phases also need to be considered. In either case, there are likely to be multiple hypotheses being tested, so optimization of power for a specific type of variant may be less helpful than a global optimization. For this purpose, we have previously considered Asymptotic Relative Cost Efficiency, a quantity inversely proportional to the total cost times the variance of the parameter of interest, combining main and substudy data (Thomas, 2007), but more recently in the context of designs for sequencing using DNA pooling, we aimed to optimize power subject to a constraint on total cost (Liang et al., 2012). We adopt a similar approach to optimize designs for testing the 1 degree of freedom Madsen and Browning (2009) rare variant burden test (Methods section Optimization of Two-phase Studies), but this could be easily extended to maximize power for multi-dimensional hypotheses. Here, we summarize a small simulation study to illustrate the potential of two-phase designs (Methods section Simulation of Two-phase Designs).

Results using the simulated sequence data are shown in Table 1 for the full cohort (the “ideal” results if the entire sample could have been sequenced) compared with two-phase analyses using (1) imputation, (2) the Horvitz-Thompson WL approach with sample weights (Horvitz and Thompson, 1952); (3) the Breslow-Cain PL (Breslow and Cain, 1988); and (4) the Breslow-Holubkov semi-parametric estimator (Breslow and Holubkov, 1997a,b). The top half of the table provides results for the Madsen-Browning index including all variants present in the parent case-control study, while the bottom half is limited to those seen at least once in the subsample; for the latter, the risk index would include different variants for the different designs, so point estimates are not comparable. The last line gives the average estimate, empirical standard deviation of estimates across replicates, and the estimated non-centrality parameter (NCP) for the Wald test if no subsampling were done. Generally, the imputation approach was the least efficient for all designs, followed closely by WL estimator, while the semi-parametric one the most efficient. Except for the WL, the optimal sampling design was also the most efficient; the inefficiency of the WL in this case seems to be due to some small strata receiving very large weights (for example, 500/2 in the low-risk case stratum compared with 500/214 in the high-risk case stratum, see Footnote b of Table 1). In earlier simulations (not shown) we found relatively little inflation in the variance estimates or changes in the point estimates as the number of strata increases (although the number of replicates that failed to converge increased). Further research in the case of many sparse strata would be helpful, as well as on such issues as the size of the region to be sequenced and the depth of sequencing.

**Table 1:** **Parameter estimates (SEs) [Wald *Z*-tests] for the simulated two-phase sequencing data using the imputation, weighted likelihood, Breslow-Cain pseudo-likelihood, and Breslow-Holubkov semiparametric maximum likelihood estimators**.

**Analysis method**	**Subsample design**		
	**Case-control**	**Balanced^a^**	**Optimal^b^**
**ALL 1422 RARE VARIANTS IN THE FULL STUDY (47 CAUSAL)**			
Imputation	1.69 (0.96) **[1.76]**	1.75 (0.86) **[2.03]**	1.63 (0.79) **[2.06]**
Weighted likelihood	1.88 (0.96) **[1.96]**	1.89 (0.91) **[2.08]**	1.72 (1.13) **[1.52]**
Pseudolikelihood	1.88 (0.96) **[1.96]**	2.03 (0.97) **[2.09]**	2.22 (1.00) **[2.22]**
Semiparametric ML	2.12 (0.98) **[2.16]**	2.22 (0.99) **[2.24]**	2.24 (1.00) **[2.24]**
Full Study	1.80 (0.69) **[2.61]**		
**VARIANTS DISCOVERED IN THE SUBSTUDY ONLY**			
Average number discovered (causal)	653 (44)	719 (45)	697 (44)
Imputation	1.66 (0.95) **[1.75]**	1.73 (0.86) **[2.01]**	1.64 (0.80) **[2.05]**
Weighted likelihood	2.34 (1.01) **[2.32]**	1.87 (0.93) **[2.01]**	1.73 (1.12) **[1.54]**
Pseudolikelihood	2.35 (1.02) **[2.33]**	2.01 (1.00) **[2.01]**	2.27 (0.97) **[2.34]**
Semiparametric ML	2.56 (1.06) **[2.42]**	2.19 (1.02) **[2.15]**	2.29 (0.97) **[2.36]**

Empirical mean estimates and standard deviations are computed from 1000 replicates with 2000 cases and 2000 controls showing association with at least one GWAS SNP, subsampling 600 subjects, 50 causal rare variants. These results are contrasted across three sampling designs. Coefficients are in units of log RR per Madsen-Browning rare variant summary index divided by 1000; for consistency across designs, all rare variants are included in the index in the top portion of the table; the bottom portion includes just those discovered in the substudy, so point estimates are not comparable across sampling methods. All estimates are adjusted for the risk index.

a 100 subjects from each of the 6 strata.

b Numbers of subjects in the subsample are fixed across replicates at (2, 20, 214) cases and (74, 116, 174) controls, stratified into 3 groups of risk index from low to high, based on overall optimization for all replicates combined.

#### Application to the WECARE study of contralateral beast cancers

To illustrate the potential yield from a sequencing substudy, we consider whole genome sequencing of a subset of 200 genetically enriched subjects, with the intent of following up a subset of discovered variants by testing their associations in a larger study of 700 cases and 1400 controls. The sample sizes used for illustration derive from the WECARE study (Bernstein et al., 2004) described above. The 201 subjects in the top part of Table S1 were selected by prioritizing young age, positive FH, cases over controls, and among cases, those who received radiotherapy (and for these, longer latency). These samples are currently being whole-genome sequenced at an average depth of coverage of 30×. We used simulation to address the following questions:

1. What is the anticipated yield of variants discovered one or more times in this sample, as a function of population MAF and RR?
2. Of those discovered, what proportion would be novel (not in the 1000 Genomes Project), what proportion would be truly causal, and both novel and causal?
3. Among the discovered variants in each category (of MAF, RR, causality, and number of times seen in each series), what is the power for testing association in the main study, after Bonferroni adjustment for the number of markers tested?
4. Putting all these together, what is the expected overall yield of novel, causal discoveries?

These calculations are described in Methods section Calculation of the Expected Yield of Single-variant Tests in the WECARE Study, based on the distribution of simulated allele frequencies and RR shown in Figure S1. In a subsample of this size, most variants with MAF >0.1% would be seen at least once, including the most causal variants (Table 2). Restricting to those seen more than once considerably reduces the number of variants prioritized, as does eliminating those never or seldom reported in the 1000 Genomes Project sample, but also eliminates most of the truly causal variants. If, however, the goal is to identify *at least some* of the novel causal variants with adequate power to test them for association in the main study, then this design might still discover something in the range of 5–20 causal variants out of the total 1600 simulated, depending on the specific criteria used for prioritization, and of course depending upon the true simulation model parameters.

**Table 2:** **Expected total number of discovered variants prioritized and expected number of these that are causal, by minimum number of copies in the sequencing sample and maximum number of copies in 1000 Genomes Project data**.

**Maximum copies in 1000GP**	**Minimum copies in sequencing sample**		
	****c** = **1****	****c** = **2****	****c** = **3****
**NUMBER OF VARIANTS PRIORITIZED**			
c′ = 0	1.5 M	113 K	10 K
c′ = 1	2.6 M	265 K	30 K
c′ = 2	3.4 M	418 K	57 K
**NUMBER OF PRIORITIZED VARIANTS THAT ARE CAUSAL**			
c′ = 0	41	34	27
c′ = 1	113	97	79
c′ = 2	192	168	140
**EXPECTED YIELD OF SIGNIFICANTLY ASSOCIATED CAUSAL**			
VARIANTS FROM SECOND STAGE^*^			
c′ = 0	0.7	1.0	1.6
c′ = 1	2.1	2.9	4.2
c′ = 2	3.8	5.1	7.0

* Bonferroni corrected α = 0.05 (i.e., in addition to these causal variants, 0.05 non-causal variants are expected to be declared significant).

We also simulated gene- and pathway-level burden tests (Methods section Simulation of Gene- and Pathway-level Prioritization in the WECARE study; Table 3). These show a modest improvement in power at the higher levels of aggregation, but power is still low with these sample sizes. The simulated causal variants are predominantly rare, so only about 16% of causal ones are even discovered in this small sample, setting an upper bound for power for single variant tests. Of the 43 discovered causal variants (on average across 100 replicates), 19 are prioritized and 3 of these are found to be significantly associated in the full study sample, for an average power of 1.1%. Of course the corresponding proportions were much smaller for null variants, yielding only 3 false positives in total out of 31 million. (The elevated “false positive” rate for single variant tests compared with the target 0.05 is due to null variants in strong LD with other causal variants.) Similar comparisons yielded 2.9, 5.7, and 4.6% power for gene-regions, genes, and pathways respectively, with type I error rates at or below the target level. The improvement at the region and gene levels probably reflects the increasing benefit from pooling similar variants, while the failure of the pathway burden test to yield even better power may be due to an increasing proportion of truly null genes or variants diluting the effect of the positive ones. These results are based on prioritization at each level at $\alpha_{1}=\sqrt{0.01/p}$ where *p* is the number of tests (variants, genes, etc. discovered in the subsample); while optimization of these values is possible, the results seem to be relatively insensitive across a broad range of choices. The specific results are somewhat more sensitive to the specific model parameters, only one being presented here, but the general patterns remained consistent across all values we considered.

**Table 3:** **Simulated results of hypotheses tested in the main study for various levels of aggregation in the planned WECARE Study; means over 100 replicate simulations**.

**Test**	**True negatives**				**True positives**			
	**Total**	**Discovered**	**Prioritized**	**Significant**	**Total**	**Discovered**	**Prioritized**	**Significant**
Pathway	87.4	87.3	0.2	0.00	12.6	12.6	1.1	0.6 (4.6%)
Gene	1016	1006	8.4	0.00	33.7	33.4	5.6	1.9 (5.7%)
Gene-region	2925	2318	32.8	0.05	97.5	79.6	12.9	2.9 (2.9%)
Single variant	31,218	6558	156	3.10	273	43.0	19.4	3.1 (1.1%)

*Based on 100 pathways with an average of 10 genes each, each gene having on average 10 exonic variants (r = 1), 20 regulatory variants (r = 2), and 30 other variants surrounding the gene (r = 3) with* σ_*P*2_ = 1.0, π_*P*_ = 0.125, σ_*G*2_ = 0.25, π_*G*_ = 0.25, σ_*V*2_ = *exp*[−η_*Rv*_ − 0.25 *ln(q*_*v*_/.01)] *where* η_1_ = −1.0, η_2_ = −1.5, η_3_ = −2.0, *logit*(π_*v*_) = ζ_*Rv*_ − 0.25 *ln(q*_*v*_/.01) *where* ζ_1_ = −0.5, ζ_2_ = −1.5, ζ_3_ = −2.5.

Sequencing is still underway but preliminary results from the first 93 samples from Table S1 suggest that the majority of subjects (mainly contralateral cases with early onset and/or family-history positive subjects) carry at least one functionally significant, clinically relevant or predicted disease-causing mutation, based on external annotation criteria including Human Genome Mutation Database (Stenson et al., 2009), ClinVar [http://www.ncbi.nlm.nih.gov/clinvar,] MutationTaster (Schwarz et al., 2010) in both known and unknown breast cancer candidate genes and pathways, and >50% carry at least two and 10% carry three or more. The next step is to see whether these variants are differentially distributed between WECARE cases and (unilateral breast cancer) controls or population rates, whether they are associated with radiotherapy (suggesting an interaction effect), and to test these variants in the full WECARE study sample.

### Family-based designs for prioritization

Several investigators have recently reported approaches to efficient selection of individuals for sequencing in family-based designs. Cheung et al. (2013) described an approach for targeted sequencing of regions exploiting already available linkage information to optimize imputation to other family members, but without using phenotype information, whereas Wang et al. (2013) described an approach for whole genome sequencing using phenotype and kinship information. We simulated various family-based designs for whole genome sequencing to address the following questions:

1. What criteria should be used to select families and members for sequencing substudies?
2. What criteria should be used to prioritize variants for subsequent association testing?
3. How do family-based designs for sequencing compare with those using unrelated individuals in terms of probability of discovering novel variants, classifying variants by their likelihood of being causal, and power for testing association with disease?

We consider a two-phase design that uses a subset of individuals from a family study chosen on the basis of their phenotypes and relationships to each other for discovery and screening, followed by association testing in the full pedigrees. If enough family-based samples are available, replication using additional family-based samples is preferable to using a different sampling scheme because one would like the spectrum of variants (e.g., MAFs and RRs) being tested in the second stage to be comparable to those discovered in the first. We compare the relative efficiency of this family-based design with a conventional two-stage case-control design with comparable costs.

We evaluated these designs by simulating 4-generation pedigrees with 22 members in each. We sampled haplotypes from the same simulated population described earlier and randomly dropped genes through the pedigrees, generating phenotypes with randomly selected rare variants as causal with the same RR distribution and retaining those pedigrees with some minimum number of cases (Methods section Simulation of Family-Based Designs). To address the first question, families with various numbers of affected individuals were ascertained, and from each of these we selected individuals to sequence in various ways (e.g., two cases of at least second-degree relationship to each other and one unaffected individual). We then tabulated the following statistics for causal and non-causal variants by the relationship of the cases to each other.

- *Rule-based criterion*: the number of families for which all affected members carry the variant and no unaffected ones did among the subset sequenced;
- *Likelihood ratio (LR) criterion*: the ratio of the retrospective likelihoods under the simulated penetrances and allele frequencies vs. the null penetrance (the average rate in the ascertained families); while similar to the lod score used in linkage analysis, here a single-locus likelihood is used to test association with a directly-observed variant, not markers in LD with an unobserved locus;
- *Bayes factor (BF) criterion*: similar to the likelihood ratio, but based on the marginal probabilities under the simulated prior distributions of penetrances and allele frequencies;
- *Score test criterion*: the score test derived from the retrospective likelihood, evaluated under the null hypothesis.

(Methods section Family-based Criteria for Prioritization of Variants). The score test was evaluated both at the single-variant and the regional level, the latter using the family-based SKAT tests (Schifano et al., 2012; Chen et al., 2013; Ionita-Laza et al., 2013; Schaid et al., 2013b). The other tests were used only for ranking variants individually. The score test essentially relates the phenotypes of the entire pedigree to the genotypes of those who have been sequenced using the inverse of the kinship matrix to weight them. If linkage information is available, then a direct test of association with imputed genotypes is possible (Cheung et al., 2013), allowing for residual phenotypic correlations

**Figure 1:**
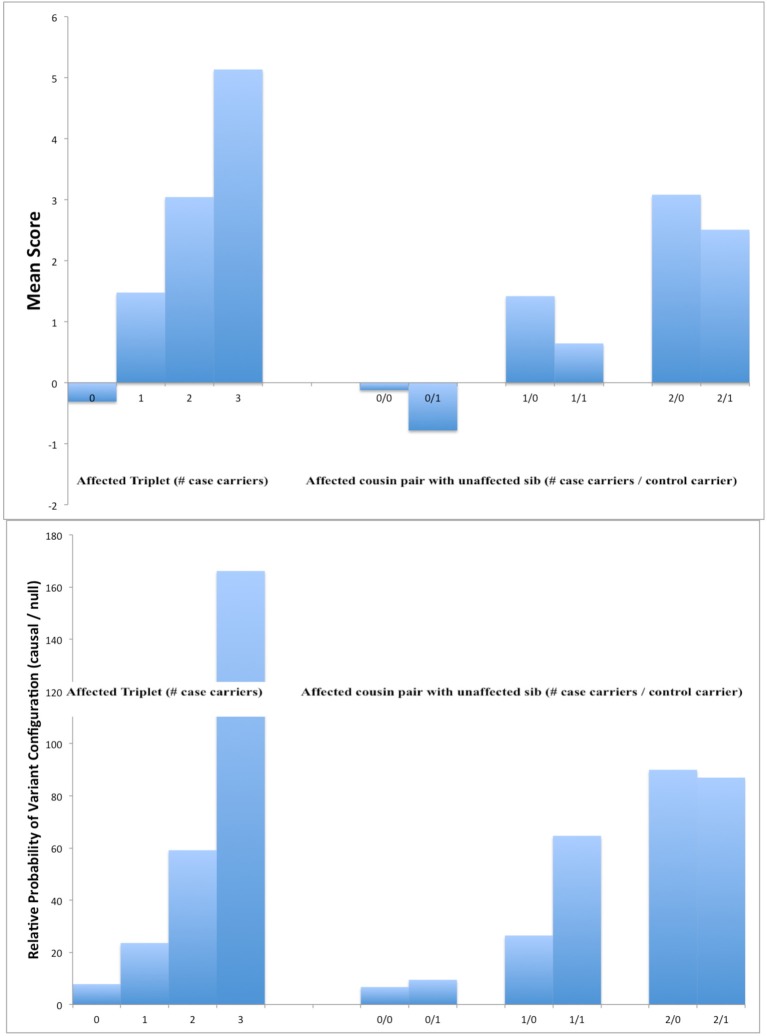
**Mean scores for causal variants (top panel) and ratio of frequencies of causal to non-causal variants (bottom panel) in simulated 11-member pedigrees with at least 4 affected members**. In each panel, results are shown for a design sequencing an affected sib pair and affected cousin by the number of carriers of the variant allele (left) or an affected first cousin pair and an unaffected sib by the number of carriers among cases and controls (right).

Figure 1 shows the mean score statistics per family for those with a total of 4 affected individuals in which either an affected sib pair with an affected first cousin or a discordant sib pair with an affected cousin have been sequenced. These were derived for an 11-member sub-pedigree for which exhaustive enumeration of all possible genotypes and phenotypes was feasible. As expected, variants with the largest scores for causal variants (top panel) and the highest probabilities of being causal (bottom panel) were those where both cases were carriers and (if sequenced) the control not. Having the control affected somewhat lowers the average score, but not as much as having an additional case being a carrier increases it, essentially because we are considering a relatively rare disease (population prevalence 5%).

**Figure 2:**
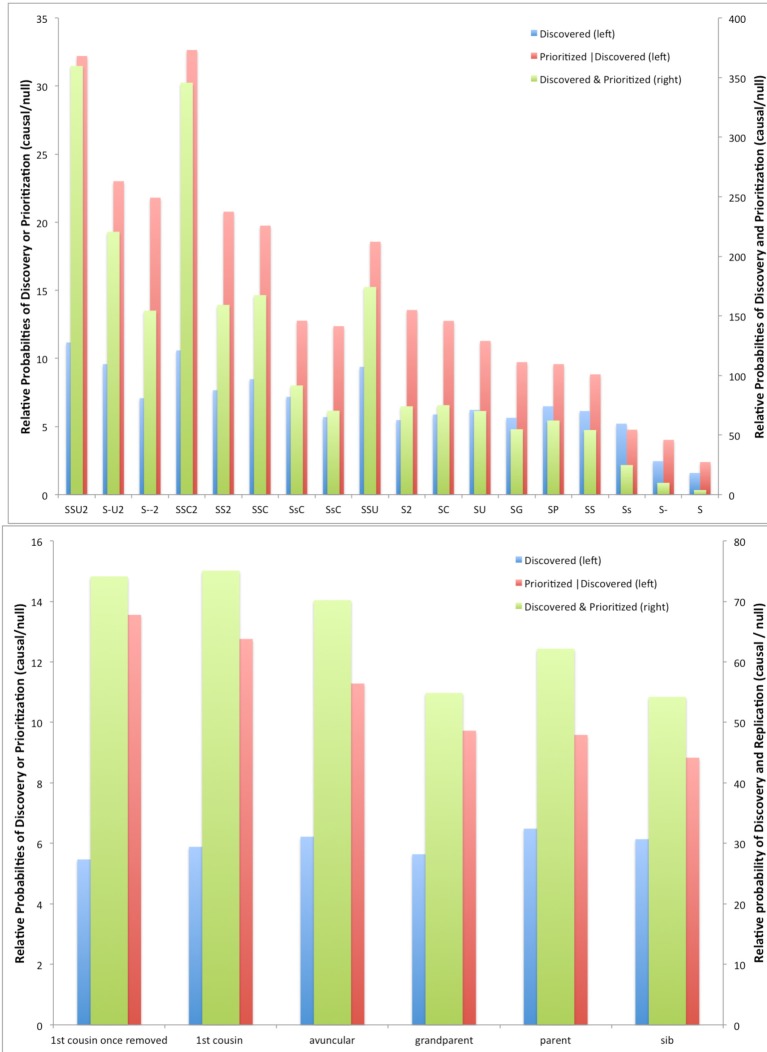
**Relative probabilities of discovery, prioritization, and both between causal vs. null variants for different criteria for selecting members for sequencing in simulated 11-member pedigrees with at least 4 affected members**. **Top panel**, all designs; **bottom panel**, detail for designs with only two members sequenced. (Codes for **top panel**: S, sib; C, cousin; 2, first cousin once removed; U, uncle; G, grandparent; P, parent; Upper case, affected, lower case, unaffected; hyphen, affected but not sequenced.)

This trade-off is explored further in Figure 2 for various types of relatives sequenced. Here, we fix the total number of individuals being sequenced at 100 across the designs being compared (e.g., 25 pedigrees with four members each sequenced, 33 with 3, 50 with 2 each, or 100 singletons). Although, as expected, having more families with fewer individuals sequenced increases the *absolute* discovery probabilities (not shown), the *relative* difference comparing causal and null variants goes the other direction, and the relative probability of prioritization also increases for more subjects per pedigree and fewer pedigrees sequenced, for a considerable increase in the overall relative probability of discovery *and* prioritization.

Comparing designs with two individuals sequenced per pedigree shows little difference in the probability of discovery across the relationships among the pairs (Figure 2), but shows that the conditional probability of prioritization given discovery and the joint probability of discovery and prioritization is better for more distant relatives.

**Figure 3:**
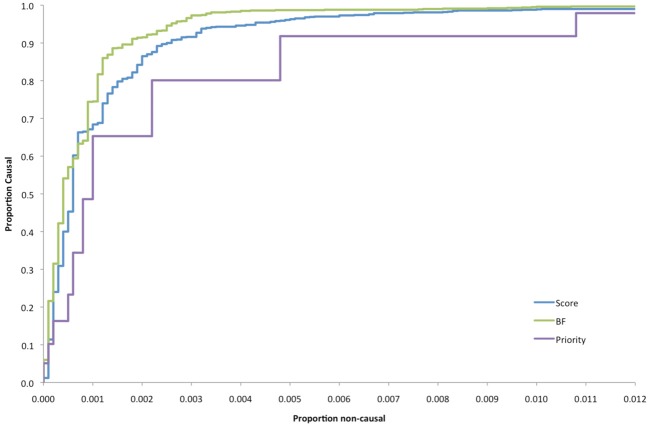
**Receiver operating curves comparing different prioritization schemes**.

Obviously, the more stringent the cutoff for any of these criteria, the fewer the variants that would be prioritized, but non-causal variants tend to be eliminated much faster than causal variants (Figure S2), so the challenge is to choose a threshold that minimizes the false positive proportion, subject to the total number of variants that can be tested in subsequent replication efforts. The relative performance of the various prioritization criteria is illustrated in Figure 3 as Receiver Operating Curves, varying these thresholds. Although the BF criterion is the best overall in terms of the area under the curve, it is the most computationally intensive and the score test is nearly as good and much faster to compute.

### Relative efficiency of family- vs. population-based designs.

We compared the power of a two-stage family-based design with that of a conventional case-control design. The overall power for any design with independent tests in the two stages is simply the product of the powers for the two stages (Methods section Calculation of Power for Two-stage Designs). The probabilities of discovery, prioritization, and replication are illustrated in Table 4 for a range of design parameters—total sample sizes, proportions allocated to stage 1, numbers of copies required to be judged a discovery, and the minimum threshold required for prioritization—under two different cost structures. (The full range of choices considered is shown in Figure S3.) Obviously, as the number required for discovery is lowered or the threshold for prioritization is raised, fewer variants in total would be prioritized, leading to a less stringent multiple comparisons penalty, but at some point the overall power decreases because too many of the truly causal variants are either not discovered or not prioritized. Although the overall power (the proportion of *all* causal variants discovered, prioritized, and replicated) for any of these designs is only about 20% (for a sample size of 1000 families), the majority of those not found are either very rare or have very small effect sizes. Of particular interest are the numbers of *novel* variants (those not in the 1000 Genomes Project database) that are discovered, prioritized, and replicated. Since these too are predominately rare, power for them is even lower, but depending upon the total number that actually exist, they could still represent a substantial yield of true positive findings.

**Table 4:** **Some near-optimal multi-stage family-based and case-control designs (The first row of each block is the one with the highest ARCE among those investigated; the second is the one with better power among those with similar costs**.)

**Total sample size^a^**	**Proportion allocated to stage 1 (%)**	**Minimum copies for discovery**	**Criterion for prioritization^b^**	**Proportion of causal discovered (%)**	**Total number prioritized^c^**	**Power for all causal all novel) variants**	**Total cost (millions)**	**ARCE^d^) (×**1000**)**
**FAMILY-BASED DESIGNS (COSTS: $1000/FAMILY, $5000/SEQUENCE, 5¢/GENOTYPE)**								
1800	30	12	3.0	38	3,591	17% (9%)	$12.3	0.252
2400	30	14	3.0	13	3,894	21% (13%)	$16.8	0.241
**FAMILY-BASED DESIGNS (COSTS: $5000/FAMILY, $1000/SEQUENCE, 5¢/GENOTYPE)**								
2100	50	12	4.0	56	346	19% (12%)	$13.8	0.264
2400	50	12	4.0	59	378	21% (13%)	$15.8	0.260
**CASE-CONTROL DESIGNS (COSTS: $100/SUBJECT, $5000/SEQUENCE, 5¢/GENOTYPE)**								
7000	20	12	0.001	62	5,502	16% (9%)	$17.8	0.171
9000	20	14	0.001	65	5,862	20% (12%)	$23.1	0.164
**CASE-CONTROL DESIGNS (COSTS: $500/SUBJECT, $1000/SEQUENCE, 5¢/GENOTYPE)**								
6000	40	16	0.0001	70	823	17% (11%)	$8.1	0.416
7000	40	16	0.0001	74	912	20% (13%)	$9.4	0.407

a Number of 22-member pedigrees for family-based designs; number of cases, number of controls for case-control designs.

b Minimum score test for family-based designs; minimum p-value for case-control designs.

c Assuming 1000 causal variants out of a total of 20 million.

d Total number of true positives, inversely weighted by the square root of MAF, divided by total cost.

These comparisons are provided for the “optimal” designs of each type and one alternative design that yields better power at modestly larger cost. Assuming costs of $1000 per family for enrollment and obtaining pedigree phenotypes, $100 per subject enrolled in a case-control design, $5000 per whole-genome sequence, and $0.05 per subject-genotype, the optimal family-based design turns out to require 540 pedigrees in stage I and 1260 in stage II at a critical value of the score test for prioritizing variants of 3.0; this yields 167 true-positive replicated associations out of 1000 simulated at a cost of $12 M. The corresponding optimal case-control design would require 1400 case-control pairs in stage I, 5600 in stage II at a critical value of α_1_ = 0.001 in stage I, for a yield of 159 true positive replicated associations at a cost of $18 M (about 2/3 the cost-efficiency of the family-based design). Of course, with different cost ratios, these optimal designs would change, as illustrated in Table 4 for the case where enrollment costs are 5 times larger and sequencing only $1000 per whole genome. In this instance, case-control designs turn out to be the more cost-efficient. For this simulated MAF distribution, only 175 of the 1000 causal variants would be novel, but in every situation considered, the power for discovering such rare variants is still more than half that for all causal variants. No striking differences were seen between the spectrum of RRs and MAFs discovered, prioritized, and replicated by the two types of designs with sample sizes chosen to yield similar overall power.

### Application to the colorectal cancer family registries data

Included in the Colorectal Cancer Family Registries are a few large families for whom all available family members have either been genotyped for previously replicated GWAS SNPs or whole exome sequence variants. We analyzed one of these—a large Australian pedigree comprising 145 individuals with a total of 7 colorectal, 1 Lynch syndrome, and 9 other cancer cases. Genotypes for 32 GWAS-associated SNPs were available for 49 of the members, including 5 of the CRC and Lynch cases and 4 of the other cancers. These data were used to illustrate the effect of subsampling. We selected individuals under various criteria, calculated LR, BF, and score statistics using only the SNP data for these selected individuals [but all the phenotype information (Visscher and Duffy, 2006)], and compared these results to those from the complete genotype data to see which criteria best distinguish variants that are “truly” associated (based on the complete data) from “false” positives.

**Figure 4:**
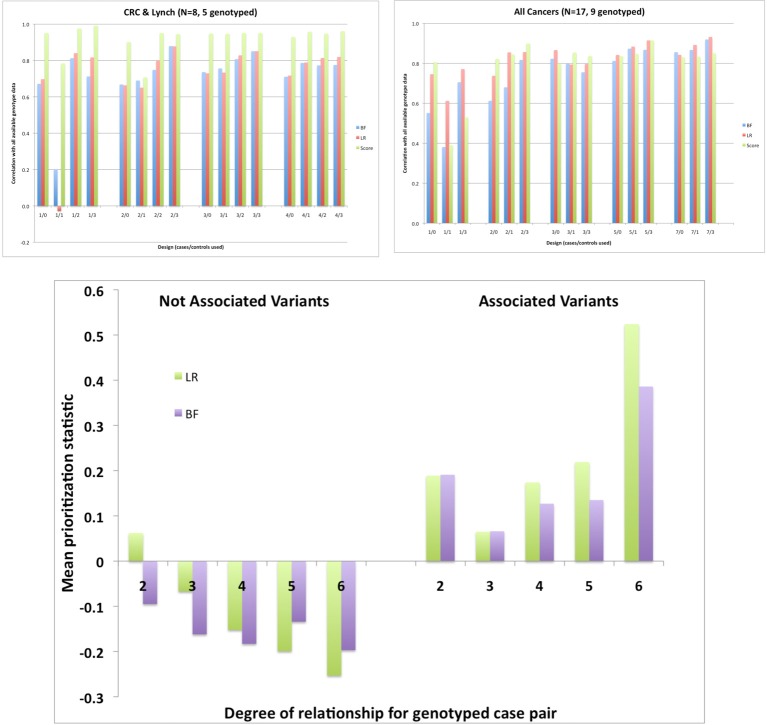
**Correlation across 32 GWAS SNPs between the statistics computed from the complete genotype data and those computed using only the genotypes for various subsets of members; top left: 5 genotyped CRC and Lynch syndrome cases; top right: 9 cases of any cancer**. Bottom: prioritization statistics by degree of relationship for apparently associated or unassociated variants based on the complete data. Data from a single 145-member Australian pedigree with a total of 8 CRC or Lynch syndrome cases and 15 cases of any cancer and a total of 49 subjects genotyped.

Figure 4 shows the correlation of each statistic computed using all available genotype data (as the “gold standard”) with those using only the subsample of genotypes (averaging over 10 replicate subsamples). For the CRC and Lynch syndrome phenotype, we compared subsets of 1–4 cases and 0–3 controls out of the 5 available cases and 44 available controls. These showed little improvement in correlation for any of the test statistics from adding more than about 2 cases. Adding the genotypes for one or two unaffected members somewhat improved the correlation for LRs and BFs when there were 2 or more cases, but surprisingly worsened the correlation when only a single case was included; this may simply reflect instability due to the small number of cases in total. Results were somewhat more stable when all 9 cancer cases with available genotypes were considered, allowing comparisons of larger subsamples of cases. Adding more than about 3 cases did not materially improve the correlation and adding 1–3 controls improved the correlations only modestly, again reducing them when a single control was added to a single case. The bottom panel shows that the more distant relative pairs were more informative about distinguishing apparently associated from non-associated SNPs (based on the complete data).

Because only common variants were available in the Australian data, we also performed similar simulations on 15 large pedigrees that had previously been included in a linkage scan (Cicek et al., 2012) and had whole exome data available on 2–3 CRC cases from each. Not surprisingly, in this small dataset, no genomewide significant associations were found by the score test with any of the 359,744 single nucleotide variants (SNVs) called at least once (a third of these were called only 3 or fewer times) or with 100-SNV bins with the regional score SKAT test, nor were the regional tests particularly correlated with the maximum single-SNV tests or with prior annotation. Additional simulations (not shown) based on the real sequence data and simulated phenotypes (conditional on the total number of cases in each pedigree) confirmed that 15 families would be far too few to find any significant causal effects, even if IBD information were used. The design of a larger NGS study is described in the concluding section.

## Discussion

One of the advantages of family-based designs is that Mendelian inconsistencies can be used to check for genotyping errors (Pompanon et al., 2005). This has, of course, long been recommended as a routine quality control check in linkage and family-based GWAS studies. This advice becomes even more important when dealing with NGS data because of its inherently higher error rate as a function of depth of sequencing and quality control filters applied (Faye et al., 2013). Further research on approaches to using pedigree information to improve variant calling would be helpful.

In a similar vein, Mendelian inheritance could be exploited for improved imputation of variants in unsequenced family members. On obvious way to proceed would be to first use standard imputation procedures with external reference populations (Howie et al., 2012), treating each subject with GWAS SNP data as independent to obtain preliminary genotype probabilities at the sequenced variants. These could then be combined with the observed genotype calls for the sequenced family members using Mendelian transmission probabilities to obtain refined genotype probabilities (Burdick et al., 2006). While proceeding variant-by-variant in this manner is relatively straightforward, it fails to take LD patterns among the variants into account, but a similar strategy could be applied to haplotypes (Cheung et al., 2013). Such a two-step approach was used in simulations for the Genetic Analysis Workshop 18 (http://www.gaworkshop.org/gaw18/index.html). Ideally, a unified approach that would integrate the two sources of information in a single step would be preferable. In addition to imputation, identity-by-descent information could be used to inform the selection of subjects for sequencing (Cheung and Wijsman, 2013) and directly as a local genetic similarity kernel in family-based SKAT tests.

Homozygosity mapping in families has proven to be a valuable technique for mapping recessive alleles (Kruglyak et al., 1995; Chahrour et al., 2012). Design issues for sequencing studies for such traits are likely to be somewhat different from those considered here and would be useful avenue for further research.

Another possibility is to use a two-step analysis of the *same* data, exploiting between-family comparisons to prioritize variants and within-family comparisons to test the most promising ones, in the spirit of Van Steen et al. (2005). Because these two tests are independent, one then need only correct the significance level for the number of variants passed to the second stage. For quantitative traits, regression of the offspring phenotypes on the mean of the parents' genotypes provides a simple first-step test. For disease traits, one would have to include control trios, nuclear families with varying proportions of affected offspring, or external control individuals to have the variability in phenotypes needed for the first-step test. Practically, however, this approach would require having access to the DNA for case-parent trios (which might not be available for late-onset diseases like cancer) and sequencing of the parents rather than the cases for the first stage; not only would this double the sequencing costs over a more conventional design that sequences only the cases, but it might seem counter-intuitive since the parents may not themselves be affected (even though at least one of each pair must carry any variant that case does). One could of course reverse the two steps, but this would require sequencing the entire trio in the first step and the final inference would not be robust to population stratification. In further simulation studies (not shown), we found that use of external controls tends to be more powerful than between-family comparisons for the first step, but is more susceptible to population stratification bias; this is not a threat to validity if used in the first step, but could reduce power if too many false-positives are passed to the second step, inflating the multiple testing penalty. The two-step analysis approach consistently yielded better power than a two-stage case-control design, however.

Two-stage and two-phase designs are also amenable to considerable cost-efficiency gains by using DNA pooling techniques (Sham et al., 2002) in the first stage, thereby allowing one to sequence many more subjects than would be feasible if one were to sequence individuals. Of course, only aggregate allele frequency information (Huang et al., 2010), not individual genotypes, are then available [unless one uses molecular bar-coding techniques (Craig et al., 2008)], but these can still be used for discovery of novel variants (Lee et al., 2011) or case-control comparisons of pool allele frequencies (Johnson, 2007; Macgregor et al., 2008; Zhao and Wang, 2009). Further cost-efficiencies are possible by constructing pools of pools, with bar-coding of the sub-pools (Smith et al., 2010). Optimization of designs using DNA pooling has been described by Liang et al. (2012), but extension to family-based studies remains a challenge (Lee, 2005).

We conclude by describing how these considerations influenced the design of a planned whole-exome sequencing study within the colorectal CFR. We are planning a three-stage family-based design, in which the first stage would use already available sequence information for prioritizing about 1000 genes. This would be followed by two stages of replication, each with probands from about 1000 multiple case families and 1000 controls. The first stage would exploit existing control data from the 1000 Genomes Project, while the second and third stages would use individually matched population controls. Because our hypothesis is that causal genes may harbor multiple rare variants—not necessarily the same across families—the two replication stages would perform full resequencing of the entire coding and flanking regions of the prioritized genes, 1000 genes in stage 2, 100 genes in stage 3. For the same reason, we have decided to use a family-based design for all three stages, since variants discovered in multiple-case families may not be well represented in unselected series of population-based cases. After analysis of the sequencing data from each stage, additional genotyping of the prioritized variants would be done on all other available family members for analyses using a conditional segregation analysis (Hopper et al., 1999). Three criteria would be used for prioritization at every stage: a family-based test of co-segregation with disease for each variant separately and for entire genes; a gene-based test of association comparing cases and controls; and filtering based on bioinformatics predictors. The first of these uniquely exploits the information available from a family-based design and can be used to rank genes on the probability they carry at least one causal variant, using an aggregate assessment of the impact of all rare variants in the gene. The three comparisons would be unified through hierarchical modeling, in which both the family-based and case-control comparisons would be incorporated in the likelihood for the first (individual)-level model, and the bioinformatics predictors would be incorporated in the second (variant)-level model. Similar issues are currently being discussed in the design of a large-scale sequencing study for the WECARE project. Since this is not a family-based study, the key decision there is how best to select the subset for sequencing in a two-phase design.

## Methods

All simulations were based on the same population of 10,000 haplotypes of length 250 Kb generated by the COSI program (Schaffner et al., 2005) with the population history parameters provided in their Table 1. This population contained 5125 unique variants, of which 4557 had minor allele frequencies (MAF) <0.05, 95% less than 0.01, 79% less than 0.001.

### Simulation of two-phase designs

We postulated a disease model involving multiple rare variants drawn from the simulated haplotype population with the probability of having any effect and the expected size of the effect depending inversely on the MAF (Figure S1). We then sampled pairs of haplotypes at random from this population, computed their risk under this model, and assigned case-control status, continuing in this manner until the target number of 1000 cases and 1000 controls were obtained for the parent GWAS, and tested these data for association with all common SNPs (MAF >5%). If a significant association is found with one or more SNPs, the replicate was retained for the sequencing substudy.

For the subsample, we first constructed a risk index based on a multiple logistic regression of disease state on all non-redundant GWAS SNPs and stratified the phase 1 subjects into three strata of high, medium, and low risk (with cutpoints at the 25th and 75th percentiles). We compared case-control, balanced, and optimal sampling of 600 subjects total out of the available 4000 (Methods Section Optimization of Two-phase Studies). For these subjects, we retained *all* variants, including the causal ones but also many more irrelevant ones.

Finally, we conducted a joint analysis of both phases. We tested association with the Madsen and Browning (2009) index—the number of rare variants weighted inversely by the square root of their allele frequencies—as the rare-variant covariate of interest, treated as continuous, using the WL, PL, and semi-parametric likelihood methods described in Methods Section Likelihoods for Joint Analysis of Two-phase Studies. We found that the risk index used for sampling was a confounder of the Madsen-Browning rare variant index, due to LD among the variants included in each, due to differences between the weights in the Madsen-Browning index and the simulated weights, and due to having used the disease status to construct the risk index, so all results were adjusted for the sampling risk index. This entire process was repeated 1000 times.

### Likelihoods for joint analysis of two-phase studies

Following the general notation used by Breslow and Holubkov (1997a,b), we let *V* represent a set of GWAS SNPs in a region found to be associated with disease *Y*, and *X* represent the causal variant(s) in LD with the GWAS SNPs, to be discovered by sequencing the region on a subsample of subjects. For this purpose, we defined the causal variable *X* to be the Madsen-Browning index. The imputation strategy entails simply fitting a regression model for *X|V* to the substudy data, and then using $\hat{X}(V)$ as the covariate for *Y|X* in the full study. Proper inference would, however, require that the uncertainty in the imputation be taken into account in the analysis of the main study data. We now describe a formal likelihood approach to accomplish this.

If the first stage is a case-control sample, then the full likelihood would be the retrospective probability

$$\begin{array}{l} L_{1}(\alpha ,\;\beta ,\;\gamma )=\mathrm{Pr}(\text{V}|\text{Y})=\prod_{i\;=\;1}^{N}\sum_{x}\mathrm{Pr}(V_{i},X_{i}=x|Y_{i})\\ \;=\prod_{i\;=\;1}^{N}\frac{p_{\gamma }(V_{i})\sum_{x}p_{\beta }(Y_{i}|x)p_{\alpha }(x|V_{i})}{\sum_{\nu }p_{\gamma }(\nu )\sum_{x}p_{\beta }(Y_{i}|x)p_{\alpha }(x|V_{i})} \end{array}$$

and the likelihood for the second stage sample S would be

$$L_{2}(\alpha ,\;\beta )=\mathrm{Pr}(X|Y,V,\;S)=\prod_{j\in S}\frac{p_{\beta }(Y_{i}|X_{i})p_{\alpha }(X|V_{i})}{\sum_{x}p_{\beta }(Y_{i}|x)p_{\alpha }(x|V_{i})}$$

The full likelihood would then be *L*(θ) = *L*_1_(α, β, γ) *L*_2_(α, β) where θ = (α, β, γ). In practice, however, both *V* and *X* are highly multidimensional and we wish to avoid having to specify their joint LD distribution parametrically. When we do not assume functional forms of *p*_α_ (*X|V*) and *p*_γ_(*V*) the likelihood above becomes suitable for semiparametric maximum likelihood (SPML) estimation. Both Breslow and Holubkov (1997a,b) and Scott et al. (2007) have developed profile likelihoods by maximizing out the high-dimensional parameters *p*_α_ (*X|V*) and *p*_γ_ (*V*), with a different parameterization. We followed a recent formulation of the problem from Scott et al., in which the estimating equations for β and the constraints for nuisance parameters π are described in a “log-likelihood”

$$\begin{array}{l} l^{*}(\beta ,\;\pi )=\sum_{y,\;v,\;x}n_{yvz}\log p_{yv}^{*}(x;\beta ,\;\pi )+\sum_{y,\;v}N_{yv}\log\pi_{yv}\\ \;-\sum_{y,\;v}n_{yv}+\log(N_{+v}\pi_{yv}-{\bar{N}}_{yv}) \end{array}$$

where π_1*v*_ = 1 − π_0*v*,_N_yv_ = *N*_*yv*_ − *n*_*yv*_, and

$$p_{1v}^{*}(x;\beta ,\pi )=\exp\text{​it}\left[x^{\prime }\beta +\log\left(N_{+v}-\frac{{\bar{N}}_{1v}}{\pi_{1v}}\right)-\text{log}\left(N_{+v}-\frac{{\bar{N}}_{0v}}{\pi_{0v}}\right)\right]$$

By iterating between maximizing a logistic likelihood with fixed offsets containing _π_ and updating _π_ using its constraint equations, we can obtain semiparametric (SPML) efficient estimates of β. The SPML approach has the advantage of being flexible about the distribution of covariates *X* and *V* while retaining good efficiency. However, the derivation of the semiparametric estimating equations was complex enough that it appeared only after two approximation methods—the WL and the PL—had been published. The WL approach weights individual score functions from model *p*_β_ (*Y|X*) inversely proportional to the sampling probabilities. In our implementation, the original Horvitz-Thompson weights *N*_*yv*_/*n*_*yv*_ were used, although an improvement might be to use predicted weights 1/Pr(*S* = 1|*Y, V, Z*) that could incorporate auxiliary information *Z* from the full cohort (say, from a logistic model), or better yet, the calibrated weights described in Breslow et al. (2009b). The PL approach was first developed in Breslow and Cain (1988), representing an alternative that uses first phase information. Following their seminal paper, we work with a PL based on *p*_β, δ_ (*Y|X, V, S*), which incorporates the parameter of interest β and the nuisance log-odds δ for *Y* = 1 in stratum *V* = *v*. They insert estimates ${\hat{\delta }}_{v}=\log(N_{1v}/N_{0v})$ into the PL and then solve for β. Schill et al. (1993) proposed to estimate δ and β simultaneously; this method, although not implemented here, has been reported to yield similar results as the Breslow and Cain (1988) version.

### Optimization of two-phase studies

Our general strategy for optimization of any of these two-phase designs aims to solve the following problem. Suppose we have collected phase I data on *N* subjects and seek to selectively assemble data on at most *n* subjects based on available information. The available information from phase I, mainly observations of *Y* and *V*, is summarized by the cell sizes *N*_*yv*_. What we wish to optimize are the cell-specific sampling fractions, denoted by *s*_*yv*_ = *n*_*yv*_/*N*_*yv*_. A natural choice of objective function might be to aim for more precise parameter estimates per unit cost, for example using the Asymptotic Relative Cost Efficiency (Thomas, 2007). However, while this goal could be readily achieved for a scalar parameter as in Reilly (1996), it is less clear when more than one parameter is estimated. In this work, we chose our objective function to be the non-centrality parameter of the likelihood ratio test for $H_{0}:\hat{\beta }=0$ vs. $H_{1}:\tilde{\beta }\neq \text{0}$, with $\tilde{\beta }$ being the subset of interest in β. This objective is equivalent to a linear combination of information matrix entries, and is thus a good summary of the standard error estimates. We denote the entire parameter vector including β and other nuisance parameters as θ. It has been shown (Self et al., 1992; Brown et al., 1999) that the non-centrality parameter can be computed as $\lambda =2E_{\theta \;A}[i(\theta_{A})-l({\hat{\theta }}_{A})]$, where *l*(.) is the log-likelihood function, θ_*A*_ is the true parameter vector under the alternative hypothesis, and ${\hat{\theta }}_{0}$ is the parameter vector that maximizes E_θ*A*_ [*l*(θ)] under the null hypothesis. A slightly different form, λ′ = λ − *v* with ν being the degrees of freedom of the test, is also reasonable. In this particular problem, we used l^*^(.) shown in the previous section in place of *l*(.). It has been shown (Scott and Wild, 1989; Scott et al., 2007) that the profile log likelihood l^*^(.) is amenable for standard likelihood ratio tests.

This problem setting requires *N*_*yv*_, Pr(*X|Y, V*), β_0_ (true value of β) to be either known or pre-specified. To obtain these quantities, we simulated 1000 data sets as described in section Simulation of Two-phase Designs, and consider these data sets as the underlying “super-population.” Then we used the estimates of Pr(*X|Y, V*), β_0_ and the average values of *N*_*yv*_ from this super-population as the input to the optimization procedure. Hence we used a fixed optimal design for all simulation replicates, using the SPML. Compared to solving the optimization problem for each replicate, this strategy represents a solution to the “expected” problem.

### Calculation of the expected yield of single-variant tests in the WECARE study

The estimates in Table 2 were derived using only the MAF distribution from the simulated haplotype population. The expected carrier probabilities in each age, FH, and disease stratum were computed from the assumed distribution of RRs over a grid of MAF and RR values using standard Mendelian inheritance methods. We computed the probability of observing *c* copies of a variant in the subsample from the Poisson distribution for each MAF and RR bin, summing the expected counts over all sampling strata, and in a similar manner, we computed the probability of observing *c*' copies among 1000 population controls. The total yield of discovered novel variants is then the sum over these bins of the number of variants in each bin times the probability of seeing >*c* and <*c*′ copies.

For association testing in the main study, we computed the NCP for the Mantel-Haenszel test (stratified by age and FH) for each bin and then computed power by reference to the cumulative normal distribution with Bonferroni correction for either the total number of discovered variants or only the number of novel variants. These are again summed over all RR and MAF bins to estimate the expected yield for various values of *c* and *c*' given in Table 2.

### Simulation of gene- and pathway-level prioritization in the WECARE study

To compare the power of single-variant and burden tests, we selected variants from the haplotypes in a multi-level fashion as follows. We defined 100 pathways *p*, each comprising 1–20 genes *g*, further subdivided into three regions *r* (e.g., “exons,” “introns and promoter regions,” and “more distant enhancer regions”). Starting and ending locations of each gene and is sub-regions were selected at random and all variants *v* within these regions were included. Pathways, genes, and variants were selected as causal with probabilities π_*P*_, π_*G*_, and π_*v*_ respectively, where π_*v*_ for variant *v* depends upon the type of region and its MAF. Thus, a variant has a causal effect only if all three levels are designated as causal. Each causal variant was assigned a log RR β_*v*_ as a sum of pathway, gene, and variant-level effects, each being the absolute value of a normal deviate with zero mean and variance σ^2^_*P*_, σ^2^_*G*_, and σ^2^_*v*_, respectively, σ^2^_*v*_ also depending upon the type of region and MAF. We drew two haplotypes at random for each potential subject and computed the genetic log RR as the sum of the β_*v*_s for each variant they carried. Subjects were assigned at random to an age stratum and then to disease (unaffected, unilateral, bilateral) and FH strata with probabilities depending upon their age and genetic RR. This process was continued until the target number of subjects in each age, FH, and disease stratum was obtained and a random subset of these was designated as the sequencing sample. In the real WECARE study, we prioritized the youngest cases, those with a positive FH, and radiotherapy subjects with the longest latency for sequencing. In this way, we selected 201 subjects out of the total of 2199 available for sequencing; the distribution of the entire study sample and the sequencing subsample by age, FH, and laterality is provided in Table S1.

For the analysis, we scanned the subsample to identify all variants seen at least twice. All single variants that were seen more frequently than expected (by an amount depending on the number of comparisons) based on the general population MAFs and similarly all pathways, genes, or regions that were seen more frequently than expected were prioritized. These are tested for case-control association in the main study, using a Cochran-Mantel-Haenzsel (CMH) test, stratified by age and FH, with Bonferroni adjustment for the number of comparisons at each level.

### Simulation of family-based designs

Family-based simulations used a fixed pedigree structure of 4 generations with two offspring in each generation for a total of 22 members in 7 nuclear families. We sampled two haplotypes at random for each of the founders from the simulated haplotype population and dropped them at random without recombination through the non-founders. As before, we chose causal variants and their RRs depending upon MAF, computed the genetic log RR as the sum of the β_*v*_s for each causal variant a subject carries, and assigned disease status accordingly, adjusting the intercept to yield a population prevalence of 5%. Families with the required number of cases (set to 4 for most of the results reported here) were retained and the process continued until 1000 such families were ascertained. Various criteria were used to select a subset of family members whose genotypes were to be retained for analysis (the “sequencing subset,” e.g., two affected individuals of at least second-degree relationship and one unaffected member), while retaining the phenotype information for the entire pedigree.

For each of the causal and a random sample of the non-causal variants, we computed the likelihood ratio, Bayes factor, and score statistics (described below) and tabulated these values for different configurations of genotypes among the sequenced members and their relationships to each other. For the rule-based prioritization, we also tabulated the number of variants found in at least *f*_min_ families and the number of these that were prioritized by having the target genotype configuration (e.g., both cases being carriers and the control not). The distributions of causal and non-causal variants for each criterion are shown in Figure S2 as a function of the threshold for prioritization; plotting one curve against the other yields the ROCs displayed in Figure 3.

### Family-based criteria for prioritization of variants

#### Rule-based criterion

Variants were classified on the basis of the number of families in which all sequenced cases carried the variant and any sequenced controls did not.

#### Likelihood ratio criterion

Following the principles described in Petersen et al. (1998), we estimated the probability that any particular variant is causal under a given genetic model by accumulating likelihood ratio contributions (comparing the likelihoods of the data under the alternative hypothesis that a particular variant is causal to that under the null hypothesis that it is not causal) across families. Letting Y denote the phenotypes of all family members (including those not sequenced), *G*^*bs*^ the observed sequence data, β_*v*_ the genetic RR and *q*_*v*_ the minor allele frequency for variant *v*, the likelihood ratio is

$$LR_{v}=\frac{\mathrm{Pr}(G_{v}^{obs}|Y;\beta_{v},q_{v})}{\mathrm{Pr}({\text{G}}_{v}^{obs}|q_{v})}=\frac{\mathrm{Pr}({\text{G}}_{v}^{obs}|\text{Y})}{\mathrm{Pr}({\text{G}}_{v}^{obs})\mathrm{Pr}(\text{Y})}$$

where Pr(*G*^*obs*^_*v*_, *Y*) = ∑_*G*^*unobs*^_*v*__Pr(*Y*|*G*_*v*_)Pr(*G*_*v*_). These calculations were done evaluating the likelihood under the simulated RR and minor allele frequency for each variant under the alternative hypothesis and under the induced marginal population risk for the null hypothesis.

#### Bayes factor criterion

The likelihood ratio criterion requires a maximization of the likelihood under the alternative hypothesis, which can be unstable for rare variants. To avoid this, Petersen et al. (1998) compute a Bayes factor by averaging over a prior distribution of MAF and RR. Bayes Factors are computed as the ratio of these marginal probabilities of the joint genotypes of the sampled individuals under the true model to that under the null. Of course, we do not know the true values of either β or *q*, or even their true probability distributions, so we used the simulated probability distributions for Pr(*q*) and Pr(β |*q*), averaging over a random samples of 100 parameter values drawn from their null and alternative distributions.

#### Score test criterion

We computed the score statistic *T*_*v*_ for a single variant *v* derived from a multivariate logistic model for the phenotypes of the entire pedigree and the genotypes of the sequenced subset as

$$\begin{array}{l} T_{v}=\Sigma_{f}t_{fv}=\Sigma_{f}{\left(Y_{f}-p_{f}1\right)}^{\prime }K_{f}^{-1}(G_{fv}-q_{fv}1)\\ \;=\Sigma_{f}[\Sigma_{i\in Nf}\Sigma_{j\in Sf}(Y_{fi}-p_{f})K_{f}^{ij}(G_{fjv}-q_{fv})] \end{array}$$

where *Y*_*f*_ is the vector of phenotypes for family *f*, *p*_*f*_ is the family-specific disease prevalence, *K*_*f*_ is the kinship matrix, *G*_*fv*_ the vector of genotypes for variant *v*, and *q*_*fv*_ is the mean of *G*_*v*_ among the sequenced members. The *G*_*fv*_ − *q*_*fv*_ deviations are set to zero for untyped individuals, but the inclusion of the kinship terms for typed-untyped pairs allows their phenotypes to contribute. This statistic has mean zero under the null hypothesis and asymptotic variance var(*T*_*v*_) = Σ_*f*_ *t*_*fv*_^2^. For the purpose of prioritizing variants we used the score test *T*_*v*_^2^/var(*T*_*v*_) for each variant and select the top-ranked ones at some cutoff. This provides a pure within-family comparison, but those families for which all sequenced individuals are no carriers or all are carriers become uninformative. A more powerful test that exploits both between- and within-family information replaces the *p*_*f*_ and *q*_*fv*_ by and, the corresponding means over all families. The regional (SKAT) test for all variants in region *R* is simply [Σ_*f*_ (Σ_*v* ∈ *R*_*t*^2^_*fv*_)]^2^/Σ_*f*_ (Σ_*v* ∈ *R*_*t*_*fv*_^2^)^2^.

### Calculation of power for two-stage designs

Using family-based simulation described above, we tabulated the average number of families in the simulated sample in which each variant was seen at least once and the NCP as the mean of the simulated score statistics by bins of MAF and RR. To compare designs with different stage 1 sample sizes and discovery thresholds, we rescaled the numbers of families carrying a given variant by the ratio of proposed and simulated sample sizes and recomputed the probability of discovery by reference to the Poisson distribution in each bin. In a similar manner, we computed the probability of prioritization at stage one at threshold λ_min_ in each bin by rescaling the NCPs by the ratio of sample sizes and referred them to the non-central chi square distribution. To extend our simulation results to the whole genome, we multiplied the predicted number of simulated null variants meeting our discovery and prioritization criteria by 20,000,000/1000. The total number of variants carried forward to stage 2 is then simply the sum over all MAF and RR bins of the product of the number of variants in the population times the probabilities of discovery and prioritization. Power for stage two was computed in a similar manner by rescaling the NCPs by the stage 2 sample size and referring it to the non-central chi square distribution with Bonferroni correction for this number of tests carried forward. Thus, the yield of causal variants discovered, prioritized, and replicated is the sum of the number of variants in the population times the probability of discovery, prioritization, and replication over all MAF and RR bins. In each MAF bin, we also computed the Poisson probability that a variant would not have been seen at least twice in 1000 population controls and computed the power for novel variants in a similar manner.

Calculations for case-control designs were similar, except that no simulation was required. The probability of discovery could be computed directly from the Poisson distribution in the combined case and control sample and the NCP s for the chi square test for allelic association computed in the usual way for a 2×2 contingency table of allele counts by case-control status.

### Conflict of interest statement

The authors declare that the research was conducted in the absence of any commercial or financial relationships that could be construed as a potential conflict of interest.

## Supplementary material

Figure S1 **Simulation parameters for models 1 and 2: top: probability of causality and mean RR for causal variants as a function of MAF; bottom: frequency distribution of non-causal and causal variants as a function of MAF**.

Figure S2 **Yield of prioritized variants as a function of the number of families required for prioritization, the minimum Bayes factor, and the minimum score**.

Figure S3 **Two-stage designs using Bayes factors for prioritization**. **Top panel**, varying number of variants required for discovery (1–4) and minimum BF for prioritization; **bottom panel**, detail of left-most portion, varying the sample size for replication *N* = 20(×2)10880. The colors indicate the proportions of simulated causal variants that are discovered, prioritized, and discovered.

Table S1 **Sample sizes used to illustrate power calculations for a two-phase design for the WECARE study (note that the actual sequencing sample is further stratified by radiotherapy and latency for the purpose of studying gene-radiation interactions, factors not considered in these simulations)**. ^*^UBC, unilateral breast cancer (controls); CBC, contralateral (second asynchronous) breast cancer (cases)
