# Supplementary figures and images for: Two-phase and family-based designs for next-generation sequencing studies
Source: Front Genet. 2013 Dec 13;4:276. doi: 10.3389/fgene.2013.00276 (PMC3861783; doi:10.3389/fgene.2013.00276)

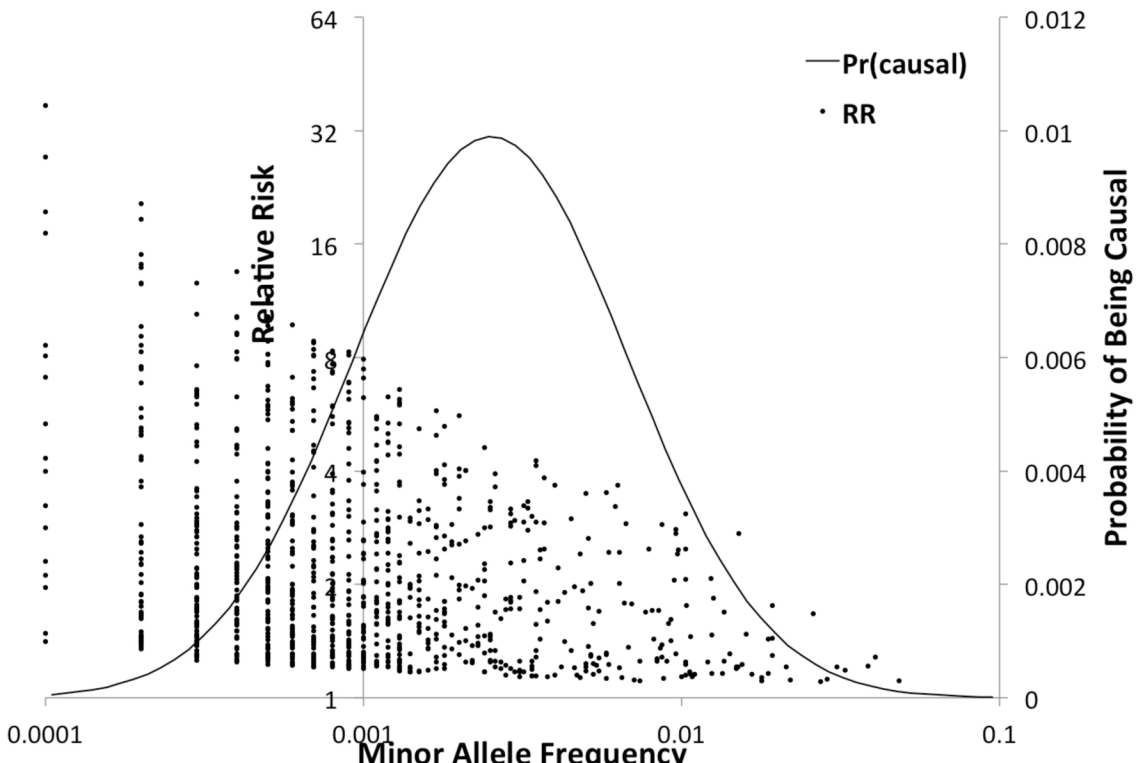

Supplement: Figure S1 — Simulation parameters for models 1 and 2: top: probability of causality and mean RR for causal variants as a function of MAF; bottom: frequency distribution of non-causal and causal variants as a function of MAF. [file Presentation1.PDF]

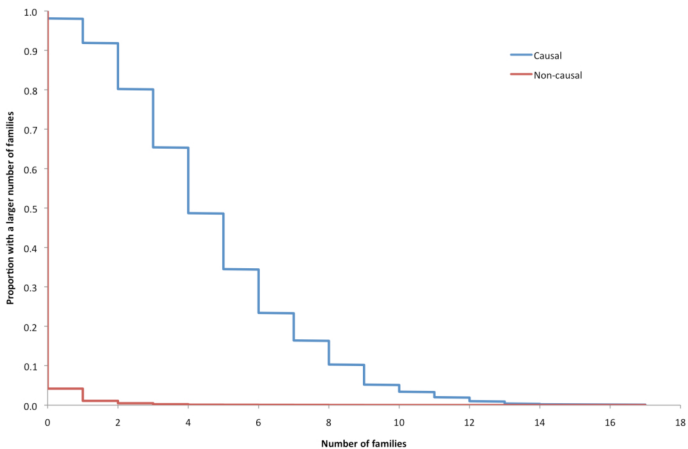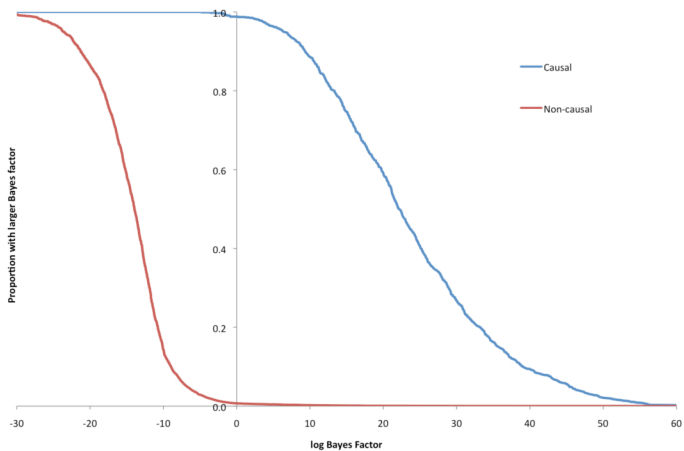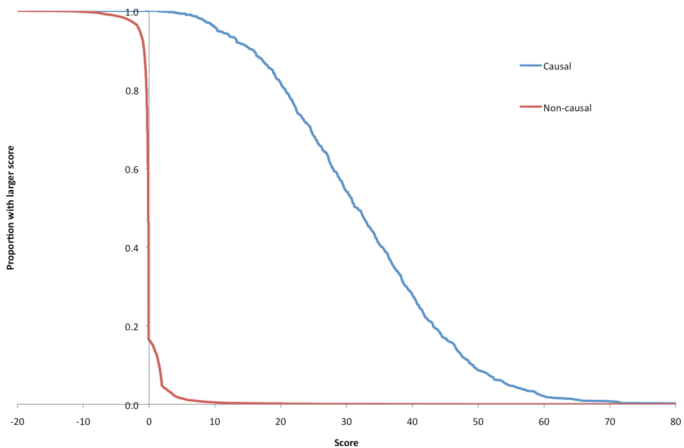

Supplement: Figure S2 — Yield of prioritized variants as a function of the number of families required for prioritization, the minimum Bayes factor, and the minimum score. [file Presentation2.PDF]

**Two-stage designs (blocks are minimum N for discovery=1,2,3,4)**

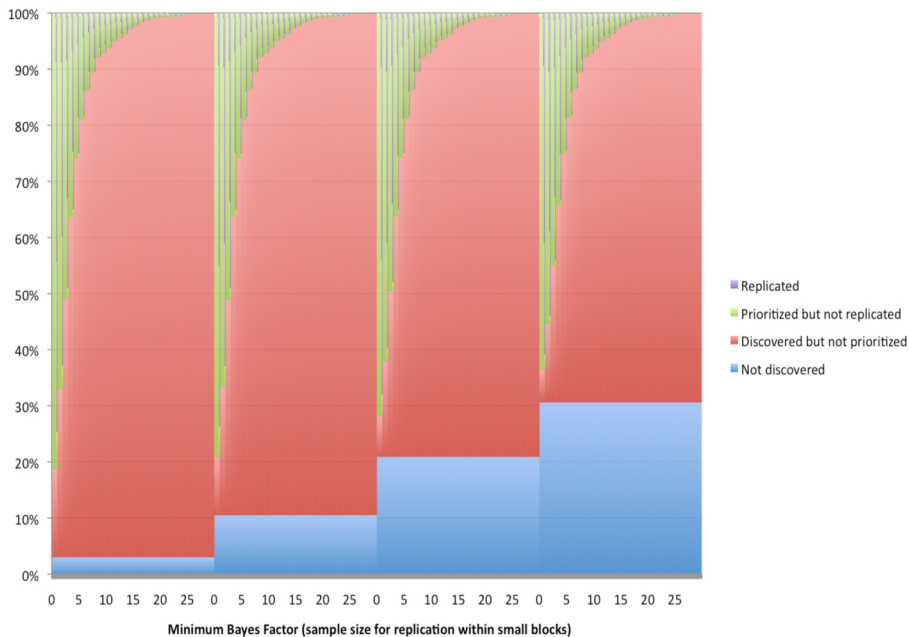

**2-stage designs (minimum N for discovery = 1)**

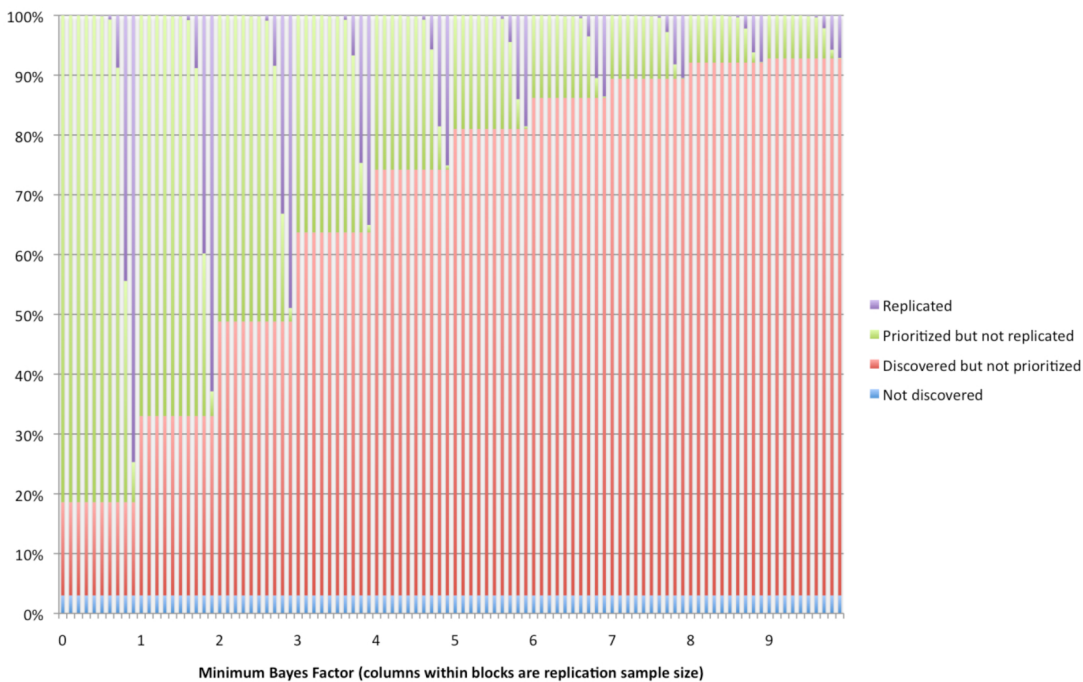

Supplement: Figure S3 — Two-stage designs using Bayes factors for prioritization. Top panel, varying number of variants required for discovery (1–4) and minimum BF for prioritization; bottom panel, detail of left-most portion, varying the sample size for replication N = 20(×2)10880. The colors indicate the proportions of simulated causal variants that are discovered, prioritized, and discovered. [file Presentation3.PDF]
