# Supplementary material for: Two-phase and family-based designs for next-generation sequencing studies
Source: Front Genet. 2013 Dec 13;4:276. doi: 10.3389/fgene.2013.00276 (PMC3861783; doi:10.3389/fgene.2013.00276)
Supplement: Table S1 — Sample sizes used to illustrate power calculations for a two-phase design for the WECARE study (note that the actual sequencing sample is further stratified by radiotherapy and latency for the purpose of studying gene-radiation interactions, factors not considered in these simulations). *UBC, unilateral breast cancer (controls); CBC, contralateral (second asynchronous) breast cancer (cases) [file DataSheet1.PDF]

	Age at first cancer	UBC Controls*		CBC Cases*	
		FH–	FH+	FH–	FH+
Sequencing Substudy	<40	15(dxage<=30)	44	41	21
	40-44	0	48	0	30
	45–49	0	0	0	2
	50+	0	0	0	0
Association study	<40	145	44	41	21
	40-44	221	48	93	36
	45–49	275	64	109	48
	50+	293	72	132	59

*UBC = unilateral breast cancer (controls)
CBC = contralateral (second asynchronous) breast cancer (cases)
